# Transgenerational transmission of aspartame-induced anxiety and changes in glutamate-GABA signaling and gene expression in the amygdala

**DOI:** 10.1073/pnas.2213120119

**Published:** 2022-12-02

**Authors:** Sara K. Jones, Deirdre M. McCarthy, Cynthia Vied, Gregg D. Stanwood, Chris Schatschneider, Pradeep G. Bhide

**Affiliations:** ^a^Biomedical Sciences, Florida State University, Tallahassee, FL 32306; ^b^Translational Science Laboratory, Florida State University, Tallahassee, FL 32306; ^c^Psychology, Florida State University, Tallahassee, FL 32306

**Keywords:** artificial sweetener, emotional behavior, intergenerational transmission

## Abstract

We report the effects of aspartame on anxiety-like behavior, neurotransmitter signaling and gene expression in the amygdala, a brain region associated with the regulation of anxiety and fear responses. C57BL/6 mice consumed drinking water containing 0.015% or 0.03% aspartame, a dose equivalent of 8 to 15% of the FDA recommended maximum human daily intake, or plain drinking water. Robust anxiety-like behavior (evaluated using open field test and elevated zero maze) was observed in male and female mice consuming the aspartame-containing water. Diazepam, an allosteric modulator of the GABA-A receptor, alleviated the anxiety-like behavior. RNA sequencing of the amygdala followed by KEGG biological pathway analysis of differentially expressed genes showed glutamatergic and GABAergic synapse pathways as significantly enriched. Quantitative PCR showed upregulation of mRNA for the glutamate NMDA receptor subunit 2D (*Grin2d*) and metabotropic receptor 4 (*Grm4*) and downregulation of the GABA-A receptor associated protein (*Gabarap*) mRNA. Thus, taken together, our diazepam and gene expression data show that aspartame consumption shifted the excitation-inhibition equilibrium in the amygdala toward excitation. Even more strikingly, the anxiety-like behavior, its response to diazepam, and changes in amygdala gene expression were transmitted to male and female offspring in two generations descending from the aspartame-exposed males. Extrapolation of the findings to humans suggests that aspartame consumption at doses below the FDA recommended maximum daily intake may produce neurobehavioral changes in aspartame-consuming individuals and their descendants. Thus, human population at risk of aspartame’s potential mental health effects may be larger than current expectations, which only include aspartame-consuming individuals.

## Significance

Exposure of mice to aspartame, an artificial sweetener found in nearly 5,000 diet foods and drinks, at doses equivalent to below 15% of the FDA recommended maximum daily intake for humans, produces anxiety-like behavior. The anxiety is alleviated by diazepam, a drug used in the treatment of generalized anxiety disorder. The aspartame exposure produces changes in the expression of genes regulating excitation-inhibition balance in the amygdala, a brain region that regulates anxiety and fear. The anxiety, its response to diazepam and the changes in amygdala gene expression are not limited to the aspartame-exposed individuals but also appear in up to two generations descending from the aspartame-exposed males.

Aspartame is an artificial sweetener widely used in low-calorie foods and drinks. Since its approval by the FDA in 1981 (1), its use has increased steadily along with controversy regarding its potential adverse effects. Today, aspartame is found in nearly 5,000 food products consumed by men, women—including pregnant women—and children, and its annual production is 3,000–5,000 metric tons worldwide (2).

Upon oral consumption, aspartame is broken down into aspartic acid, phenylalanine, and methanol (3–6), all of which can have potent effects on the central nervous system (5–12). Phenylalanine is a precursor of the monoamine neurotransmitters. Therefore, research on aspartame’s neurobehavioral effects has focused on monoamine neurotransmitter signaling. However, there is no consensus in the clinical or preclinical literature regarding aspartame’s effects on brain monoamine content or behaviors such as memory or depression (7, 8, 13–23). Similarly, despite reports of aspartame’s carcinogenic (24, 25) and metabolic side effects (26–30), a consensus on these topics is lacking. The US government policy and publications indicate that aspartame is safe when consumed within FDA recommended maximum daily intake value (DIV) of 50 mg/kg (1, 31–33).

We found that mice consuming aspartame in drinking water at a dose equivalent to approximately 15% of the FDA-approved maximum DIV for humans showed robust, dose-dependent anxiety. The anxiety was alleviated by a single administration of diazepam, a positive allosteric modulator of the GABA-A receptor, consistent with the key role of GABA-A receptors in the regulation of anxiety (34). RNA sequencing demonstrated significant changes in expression of genes associated with glutamatergic and GABA receptor signaling in the amygdala, a brain region associated with regulation of anxiety (34–37). Perhaps even more strikingly, the anxiety phenotype, its response to diazepam, and changes in amygdala gene expression were transmitted from the aspartame-exposed male founders to their descendants.

## Results

### Aspartame Delivery via the Drinking Water.

Since aspartame is consumed orally, via consumption of low-calorie foods or drinks, we administered aspartame to adult C57BL/6 male and female mice in drinking water. We used 0.03%, 0.015%, or 0.0075% aspartame based on previous reports (13, 19, 38). Since preference or aversion to aspartame can influence the total aspartame consumption, and since there is variability in aspartame preference within the B6 strain (39), we performed a two-bottle choice assay of aspartame preference. Male and female mice received free access to two drinking water bottles, one with 0.03% or 0.015% aspartame, and the other with plain drinking water. Mice neither showed preference nor aversion to 0.03% or 0.015% aspartame (*SI Appendix*, Table S1). Since aspartame consumption is associated with metabolic effects including weight gain (27, 40–42), at weekly intervals we analyzed body weight of mice consuming plain drinking water or aspartame-containing drinking water (0.03% or 0.015%; for 12 wk in males and 6 wk in females; *SI Appendix*, Table S2). We did not find a significant effect of the type of drinking water consumed [Repeated Measures two-way ANOVA; males; F_(__2,__21__)_ = 2.29, *P* > 0.05; females; F_(__2,__21__)_ = 0.99, *P* > 0.05], but there was a significant effect of time [males; F_(2,4.4)_ = 240.6, *P* < 0.0001; females F_(__2,__36__)_ = 590.4, *P* < 0.0001] reflecting normal weight gain over the 12-wk period. The interaction between drinking water and time was not significant [males: F_(22, 231)_ = 2.1, *P* > 0.05; females: F_(10,105)_ = 0.44, *P* ≥ 0.05] suggesting that the weight gain over time was not affected by the drinking water. Daily exposure to 0.03% or 0.015% aspartame for 18 wk did not produce significant changes in metabolic biomarkers in male mice (*SI Appendix*, Table S3), further suggesting lack of metabolic effects. Although we did not collect body weight data for female mice at weekly intervals beyond 6 wk, we compared body weights of female mice consuming plain drinking water or aspartame-containing water at the end of the 12-wk exposure period. There was no significant effect of aspartame exposure on body weight between plain drinking water group and 0.03% or 0.015% aspartame groups [Mean ± SEM (g); 0.03% aspartame: 27.10 ± 0.45; 0.015% aspartame = 26.84 ± 0.47; plain drinking water = 27.53 ± 0.35; one-way ANOVA, F_(__2,__21__)_ = 0.66, *P *> 0.05].

Average daily total water consumption was 7.45 ± 0.06 mL (Mean ± SEM) for males and 7.05 ± 0.07 mL for females. Based on body weight and water consumption data, we estimate that on average, male mice (average body weight 25.7 ± 0.4 g; *SI Appendix*, Table S2) received 86.4 mg/kg (0.03% aspartame group), 43.2 mg/kg (0.015% aspartame group), or 21.6 mg/kg (0.0075% aspartame group) aspartame per day, whereas female mice (average body weight 22.4 ± 0.3 g; *SI Appendix*, Table S2) received 95.5 mg/kg (0.03% aspartame group), 47.8 mg/kg (0.015% aspartame group), or 23.9 mg/kg (0.0075% aspartame group) aspartame per day. The FDA recommended maximum DIV for aspartame for humans is 50 mg/kg (33). Based on allometric conversion utilizing pharmacokinetic and body surface area parameters (43), the mouse equivalent of the human DIV is 615 mg/kg/d. Therefore, the male mice received a daily aspartame dose equivalent to 14.0%, 7.0%, and 3.5% of the FDA recommended human DIV, and the females received a dose equivalent to 15.5%, 7.7%, and 3.9% of the human DIV. The estimated average daily aspartame intake in humans is 4.1 mg/kg (44), corresponding to a mouse equivalent dose of 50.68 mg/kg (43), which was the dose delivered to mice consuming 0.015% aspartame.

### Anxiety-Like Behavior.

**Fig. 1.**
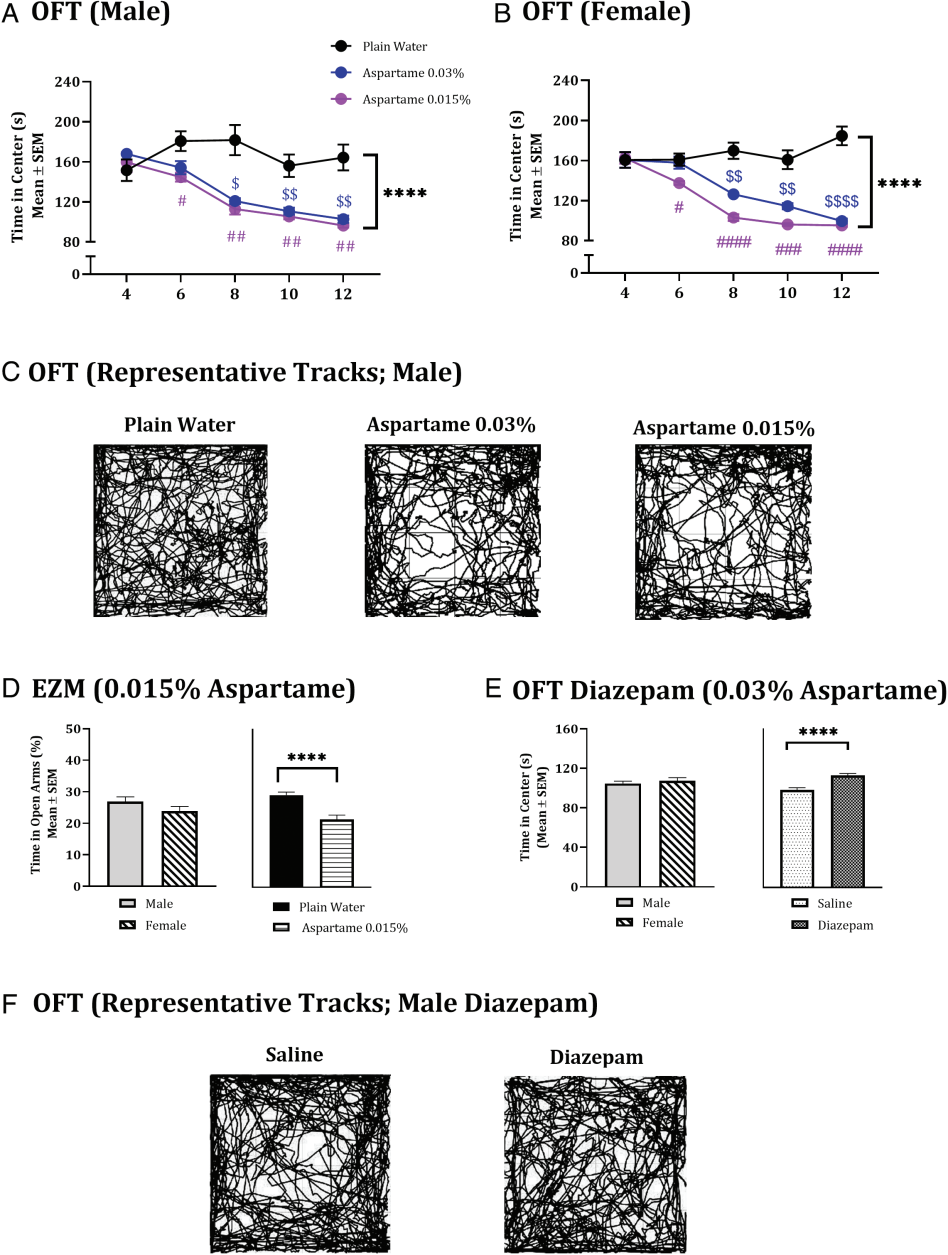
Anxiety and its response to diazepam in mice exposed to aspartame-containing drinking water. Anxiety-like responses were analyzed in male and female mice exposed daily to drinking water containing 0.03% aspartame, 0.015% aspartame or to plain drinking water for 12 wk using open field test (OFT; *A* and *B*) and elevated zero maze (EZM; *D*). In the OFT analysis (*A* and *B*), two-way ANOVA showed that male (*A*) and female (*B*) mice in the 0.03% aspartame group (blue line) and 0.015% aspartame (purple line) groups spent significantly shorter time in the center areas in the OFT compared to their counterparts in the plain drinking water (black) group (**** in *A* and *B*). Dunnett’s multiple comparisons test showed that significant differences emerged between 0.015% aspartame and plain water groups at 6 wk in males and females and persisted at 8 wk, 10 wk, and 12 wk (*A* and *B*). Significant differences between 0.03% aspartame and plain water groups emerged at 8 wk in males and females and persisted at 10 wk and at 12 wk (*A* and *B*). Typical tracks of open field exploration by one male mouse in each of the plain water, 0.03% aspartame and 0.015% aspartame groups showing differences in exploration of the center areas (*C*). The male and female mice in the 0.015% aspartame and plain water groups were examined in the EZM (*D*). Two-way ANOVA of the EZM data showed no significant effect of sex (*E*). Therefore, the data from male and female mice were analyzed together. The aspartame group spent significantly shorter time in the open areas of the EZM (*E*). Response of male and female mice in the 0.03% aspartame group to diazepam was analyzed in the OFT (*E*). Initially, baseline parameters were established 30 min following a single intraperitoneal administration of saline. Next, 48 h. later, the same mice received diazepam (3 mg/kg, i.p.) and 30 min following the diazepam administration, the mice were re-examined in the OFT (*E*). Repeated Measures ANOVA showed no significant effect of sex (*E*). Therefore, data from male and female mice were combined and analyzed. The time spent in the center areas was significantly increased following the diazepam administration compared to the saline administration at baseline (*E*). Typical tracks of open field exploration by one male mouse each in the saline and diazepam groups (*F*). Notes on symbols: # = comparison between 0.015% aspartame and plain water group; $ = comparison between 0.015% aspartame and plain water group. #; $ = *P *< 0.05; ##; $$ = *P *< 0.01; ###; $$$ = *P *< 0.001; ****, ####; $$$$ = *P *< 0.0001.

As a first step toward assessing behavioral effects of different doses of aspartame, we analyzed anxiety in the open field test (OFT). In this test, when given free access to an open field, the proportion of the total test time spent in the center areas of the open field (25% of the total area, which was 40 × 40 cm^2^) is a measure of anxiety (45). Mice with anxiety spend relatively shorter time in the center areas than mice without anxiety. We performed the OFT at 2-wk intervals beginning at 4 wk of the 12-wk period of exposure to 0.03% or 0.015% aspartame. The time spent in the center areas in males showed significant effects of drinking water treatment [Fig. 1*A*; asterisks; aspartame < plain drinking water; Repeated Measures ANOVA; F_(2,21)_ = 43.86, *P* < 0.0001], duration of exposure [F_(2.79,58.48)_ = 15.71, *P *< 0.0001], and the interaction between drinking water treatment and exposure duration [F_(__8,__84__)_ = 5.15 *P *< 0.0001]. Significant differences between 0.03% aspartame and plain drinking water groups emerged for the first time at 8 wk [Fig. 1*A*; aspartame < plain drinking water; Dunnett’s multiple comparisons test; *q*_(7.5)_ = 3.98, *P* < 0.01] and persisted at 10 wk [*q*_(8.36)_ = 3.88, *P* < 0.01] and 12 wk [*q*_(8.05)_ = 4.61, *P *< 0.01]. Significant differences between 0.015% aspartame and plain drinking water groups emerged at 6 wk [Fig. 1*A*; aspartame < plain drinking water; *q*_(9.79)_= 3.35, *P< *0.05] and persisted at 8 wk [*q*_(8.77)_ = 4.31, *P *< 0.01], 10 wk [*q*_(7.95)_= 4.37, *P* < 0.01] and 12 wk [*q*_(7.36)_ = 5.21, *P* < 0.01].

A visual inspection of typical tracks of open field exploration by one male mouse per group in plain water, 0.03% aspartame and 0.015% aspartame groups (Fig. 1*C*), reveals differences among the three groups in exploration of the center areas.

Consistent with the findings in male mice, the time spent in the center areas in the OFT in female mice also showed significant effects of drinking water treatment [Fig. 1*B*; aspartame < plain drinking water; Repeated Measures ANOVA; F_(__2,__21__)_ = 91.85, *P* < 0.0001], duration of exposure [F_(3.07,64.53)_ = 23.87, *P* < 0.0001], and the interaction between drinking water exposure duration [F_(__8,__84__)_ = 13.24, *P *< 0.0001]. Significant differences between 0.03% aspartame and plain water groups emerged at 8 wk [(Fig. 1*B*; aspartame < plain drinking water; Dunnett’s multiple comparisons test; *q*_(8.87)_ = 5.02, *P *< 0.01] and persisted at 10 wk [*q*_(8.44)_ = 4.71, *P* < 0.01] and 12 wk [*q*_(7.91)_ = 8.87, *P* < 0.0001]. Significant differences between the 0.015% aspartame and plain drinking water groups emerged (Fig. 1*B*) at 6 wk [Aspartame < plain drinking water; *q*_(8.33)_ = 3.68, *P* < 0.05] and persisted at 8 wk [*q*_(9.41)_ = 7.57, *P* < 0.0001], 10 wk [*q*_(7.29)_= 6.81, *P* < 0.001] and 12 wk [*q*_(7.48)_ = 9.46, *P* < 0.0001].

Thus, the anxiety (reduced time in the center area) emerged at 6–8 wk, depending on the aspartame concentration, and persisted until 12 wk.

We analyzed data from male and female mice separately because our goal here was to examine transgenerational transmission of aspartame-induced anxiety via the paternal lineage rather than the maternal lineage. Therefore, characterizing the time of onset of the anxiety in the founder generation of males was an essential first step.

To examine whether aspartame-induced changes in exploratory behavior or locomotion in the OFT may have contributed to the changes in time spent in the center areas, we analyzed the total distance traveled in the OFT (*SI Appendix*, Fig. S1 *A* and *B*). It did not show significant effects of drinking water treatment [Repeated Measures ANOVA; males: F_(__2,__21__)_ = 0.99, *P* > 0.05; females: F_(__2,__21__)_ = 0.07, *P *> 0.05), time [males: F_(3.98,83.76)_ = 0.7, *P* > 0.05; females: F_(3.38,70.99)_ = 1.87, *P* > 0.05), or the interaction between treatment and time [males: F_(__8,__84__)_ = 1.42, *P *> 0.05; females: F_(__8,__84__)_ = 0.16, *P *> 0.05]. Therefore, changes in exploration or locomotion did not contribute to the measurement of anxiety.

To further examine the anxiety phenotype, we tested the mice in an elevated zero maze (EZM) at the end of the 12-wk drinking water treatment. The EZM is more anxiogenic than the OFT because it incorporates two anxiogenic components, namely elevation and open unprotected spaces (46). The OFT utilizes the open, unprotected spaces but not elevation. In the EZM, mice with an anxiety phenotype spend significantly shorter time in the open areas compared to the closed areas. Neither the male nor the female mice in the 0.03% aspartame group could perform the EZM test, as they failed to meet inclusion criteria [took > 90 s to enter a closed area for the first time and made <2 total entries into the open or closed areas of the maze], likely due to a severe anxiety phenotype, which did not interfere with performance in the less anxiogenic OFT. In contrast, male and female mice in the 0.015% aspartame group met the inclusion criteria for EZM. In this group, the time spent in the open areas of the EZM showed a significant effect of drinking water treatment [Fig. 1*D*; aspartame < plain drinking water; two-way ANOVA; F_(__1,__28__)_ = 23.11; *P *< 0.0001]. The effects of sex [Fig. 1*D*; F_(__1,__28__)_ = 3.61, *P *> 0.05] or the interactions between treatment and sex [F_(__1,__28__)_ = 0.04; *P *> 0.05] were not significant, suggesting that the aspartame exposure produced comparable effects on male and female mice. The total number of area entries, a measure of exploration and locomotion, similar to the total distance traveled in the OFT, did not show significant effects of drinking water treatment [*SI Appendix*, Fig. S1*C*; two-way ANOVA; F_(__1,__28__)_ = 0.56, *P* > 0.05], sex [F_(__1,__28__)_ = 0.11, *P *> 0.05], or interaction between sex and drinking water treatment [F_(__1,__28__)_ = 0.78, *P* > 0.05].

The time spent in the open areas of the EZM was not significantly different between male mice exposed to 0.0075% aspartame-containing or plain drinking water [*SI Appendix*, Fig. S1*D*; unpaired *t* test; *t*_(14)_ = 0.47, *P *> 0.05]. The total number of area entries was not significantly different between male mice exposed to 0.0075% aspartame or plain drinking water [*SI Appendix*, Fig. S1*D*; unpaired *t* test; *t*_(14)_ = 0.57, *P *> 0.05]. Female mice exposed to 0.0075% were not examined in the EZM.

In summary, male and female mice in the 0.03% and 0.015% aspartame groups showed anxiety-like behavior in the OFT and/or EZM assays. The mice in the 0.0075% aspartame exposure were not anxious (in the EZM assay, males only). The EZM data suggest that exposure to 0.03% aspartame produced more severe anxiety-like behavior than exposure to 0.015% aspartame, whereas 0.0075% aspartame did not produce anxiety, demonstrating a dose-dependent effect of the aspartame exposure.

### Response to Diazepam.

Anxiety is associated with downregulation of GABA-A receptor signaling, and it is alleviated by diazepam, a positive allosteric modulator of GABA-A receptor in humans (generalized anxiety disorder) and rodents (34, 37, 47–51). Therefore, we examined the effect of diazepam on time spent in the center areas in the OFT in male and female mice exposed to 0.03% aspartame for 12 wk. The time spent in the center areas in the OFT was examined at baseline 30 min following a single administration of the saline vehicle. The same mice were examined in the OFT 48 h. later, 30 min following a single administration of diazepam (3 mg/kg; i.p.). There was a significant effect of diazepam on time spent in the open areas in the OFT [Fig. 1*E*; diazepam > saline; two-way Repeated Measures ANOVA; F_(__1,__28__)_ = 29.22, *P* < 0.0001] suggesting alleviation of the anxiety by diazepam. The effects of sex [Fig. 1*E*; F_(__1,__28__)_ = 1.13; *P *> 0.05] or the interaction between diazepam and sex [F_(__1,__28__)_ = 0.19, *P* > 0.05] were not significant, suggesting that diazepam produced comparable effects in males and females.

A visual inspection of typical tracks of open field exploration by a male mouse in each of the saline and diazepam groups (Fig. 1*F*) reveals differences between the groups in exploration of the center areas.

The total distance traveled in the OFT did not show significant effects of diazepam [*SI Appendix*, Fig. S4*A*; two-way Repeated Measures ANOVA; F_(__1,__28__)_ = 0.00007, *P* > 0.05], sex [F_(__1,__28__)_ = 0.03, *P* > 0.05], or the interaction between sex and diazepam [F_(__1,__28__)_ = 0.01, *P* >0.05]. When male and female mice exposed to plain drinking water were subjected to the same analysis, there were no significant effects of diazepam, sex, or the interaction between the two for time spent in the center areas [two-way ANOVA; diazepam F_(__1,__28__)_ = 0.07, *P* > 0.05; sex F_(__1,__28__)_ = 2.8, *P *> 0.05; interaction F_(1,__28)_ = 0.27, *P* > 0.05], or the total distance traveled [diazepam F_(__1,__28)_ = 0.03, *P* > 0.05; sex F_(__1,__28)_ = 0.43, *P *> 0.05; interaction F_(__1,__28)_ = 0.31, *P* > 0.05] in the OFT (*SI Appendix*, Fig. S5 *A* and *D*).

The 3 mg/kg dose of diazepam was chosen because a pilot study using 2, 3, and 4 mg/kg doses demonstrated that the 3 mg/kg dose was the maximum dose that did not produce sedative effects (i.e., reductions in distance traveled in the OFT) in male or female mice exposed to plain drinking water (*SI Appendix*, Table S4).

Repeated testing of mice in the OFT at 48 h. interval may influence performance due to habituation. A previous report showed that habituation to the OFT was not a factor when mice are tested repeatedly (52). To verify applicability of these findings to our model, we subjected male mice from the plain drinking water group to OFT twice at 48 h. interval. There was no significant difference between the initial and the second OFT in time spent in the center areas [sec.; OFT#1, Mean ± SEM= 180.60 ± 2.98; OFT#2, 181.22 ± 3.60; paired *t* test; t(4) = 0.99, *P* > 0.05] or the total distance traveled [m; OFT#1; Mean ± SEM = 20.60 ± 1.21; OFT#2, 21.40 ± 1.29; paired *t* test t(4) = 0.84, *P* > 0.05)], suggesting that the mice did not show habituation.

### Amygdala Gene Expression.

**Fig. 2.**
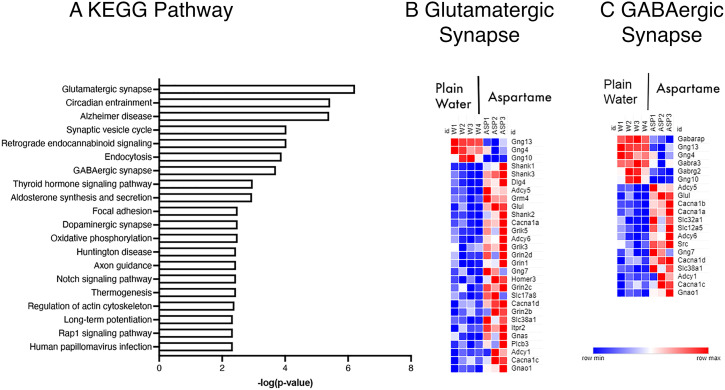
Transcriptome profiling of the amygdala by RNA sequencing in male mice exposed to 0.03% aspartame or plain drinking water for 12 wk. The top 20 most significantly enriched KEGG pathways are shown in order of lowest to highest adjusted *P* value, which is represented as the ^–^log of the adjusted *P* value on the X-axis (*A*). Heat maps showing a comparison of normalized differential gene expression values of the 30 DEGs between the aspartame and plain water groups from the glutamatergic synapse pathway (*B*) and 20 DEGs from the GABAergic synapse pathway (*C*). Rows represent the gene symbol, and columns represent each replicate. The minimum to maximum expression is indicated by a gradient from dark blue to dark red, respectively.

The effects of diazepam on aspartame-induced anxiety prompted examination of the potential mechanisms associated with excitation-inhibition (i.e., glutamate-GABA signaling) in the anxiety circuitry of the brain. We focused on the amygdala, because excitation-inhibition equilibrium in this brain region plays a central role in the regulation of anxiety (34, 53, 54). We used RNA sequencing to perform an unbiased, transcriptome-wide, discovery analysis. We found that the aspartame exposure produced 1,467 differentially expressed genes (DEGs) in male mice exposed to 0.03% aspartame for 12 wk. Among the DEGs, 1,073 were up-regulated and 394 were down-regulated. KEGG biological pathway analysis of the DEGs showed glutamatergic synapse at the #1 position and GABAergic synapse at the #7 position among the top 20 most significantly enriched pathways. (Fig. 2*A*).

Since the diazepam data prompted a focus on glutamate-GABA signaling, we examined gene expression in these two KEGG pathways further. In the significantly enriched glutamatergic synapse KEGG pathway, 30 DEGs were identified. Among the 30 DEGs, three were down-regulated and 27 were up-regulated in the aspartame-exposed group compared to the plain drinking water group. Similarly, 20 DEGs were found in the significantly enriched GABAergic synapse KEGG pathway. Among these, six DEGs were down-regulated and 14 were up-regulated in the aspartame-exposed group.

Among the down-regulated DEGs, three inhibitory guanine nucleotide protein (G_i/o_ protein) binding genes were common to both the glutamatergic and the GABAergic pathways (Fig. 2 *B* and *C*). These were *Gng13* (G protein subunit gamma 13), *Gng4* (G protein subunit gamma 4), and *Gng10* (G protein subunit gamma 10). Among the up-regulated DEGs, *Adcy5, Adcy6,* and *Adcy1* (adenylate cyclase 5, 6, and 1, part of the G protein-cyclin AMP signaling) were shared by both the glutamatergic and GABAergic synapse pathways. Thus, aspartame exposure produced significant effects on G_i/o_ intracellular second messenger pathway in the amygdala. Other up-regulated DEGs common to glutamatergic and GABAergic synapse pathways included *Glul* (glutamate-ammonia ligase, a key enzyme in the biosynthesis of glutamate and GABA) *Cacna1A*, *Cacna1C,* and *Cacna1D* (calcium voltage-gated channel subunit genes that regulate ionotropic receptor signaling).

Among the up-regulated DEGs in the glutamatergic pathway, *Grm4* (Glutamate metabotropic receptor 4, presynaptic) was at position #8, *Grik5* (Glutamate Ionotropic Receptor Kainate Type Subunit 5, presynaptic) at #12, and *Grin2d* (NMDA receptor 2D, postsynaptic) at #15 (Fig. 2*B*). All three members of the SH3 and multiple ankyrin repeat domains (Shank) family, namely *Shank1*, *Shank2,* and *Shank3,* located at the glutamatergic postsynaptic terminal were also up-regulated (Fig. 2*B*).

Among the down-regulated genes in the GABAergic synapse pathway were three postsynaptic GABA-A receptor associated genes. These were *Gabarap* (GABA-A receptor associated protein) at the #1 position, *Gabra3* (GABA-A subunit alpha 3) at the #4 position, and *Gabrg2* (GABA-A subunit gamma 2) at the #5 position (Fig. 2*C*).

Thus, an unbiased transcriptome-wide analysis identified G protein, glutamate, and GABA signaling as targets of the aspartame exposure.

**Fig. 3.**
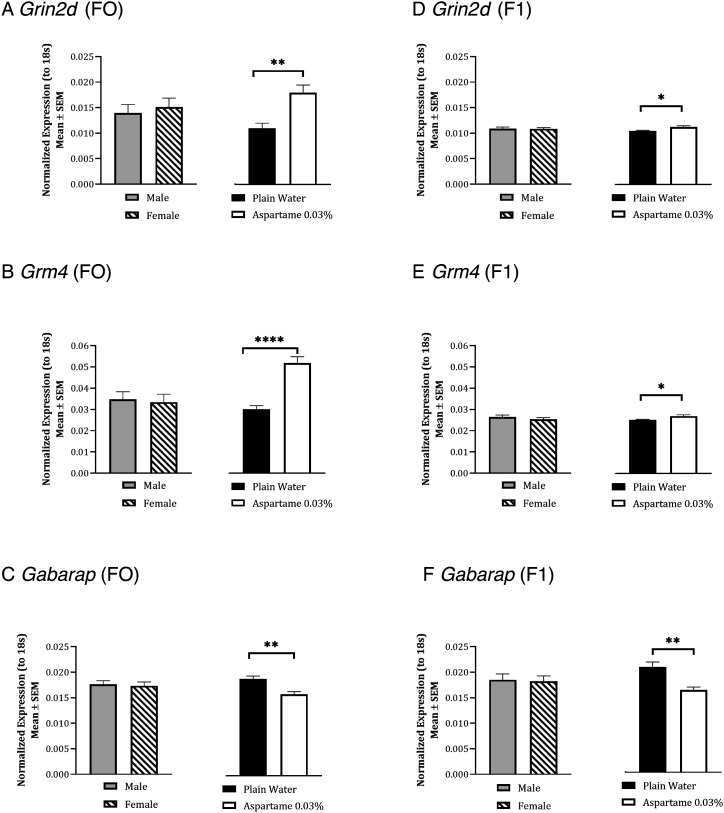
Quantitative PCR analysis of expression of mRNAs for genes in the glutamatergic synapse and GABAergic synapse KEGG pathways in F0 (*A*–*C*) and F1 (*D*–*F*) generations in the amygdala of male and female mice exposed to 0.03% aspartame or plain water for 12 wk. In each analysis, expression of the mRNA of interest was normalized to that of ribosomal 18s RNA. Two-way ANOVA did not show significant effect of sex any of the analyses. Therefore, data from male and female mice were combined and analyzed. There was a significant upregulation of mRNA for glutamatergic genes *Grin2d* and *Grm4* in the F0 (*A* and *B*) and F1 (*D* and *E*) generations and significant downregulation of the GABA-A receptor associate gene *Gabarap* in the F0 (*C*) and F1 (*F*) generations in the aspartame group. * = *P *< 0.05; ** = *P *< 0.01; **** = *P *< 0.0001.

Since the RNA sequencing was limited to male mice, we used quantitative PCR to verify changes in the expression of two glutamatergic synapse and one GABAergic synapse-related genes in amygdala samples from male and female mice. The expression of mRNA for *Grin2d* and *Grm4* showed a significant effect of drinking water treatment [Fig. 3 *A* and *B*; aspartame > plain drinking water; two-way ANOVA; *Grin2d*, F_(1,16)_ = 45.59, *P *< 0.01; *Grm4*, F_(1,16)_ = 69.2, *P *< 0.0001] but neither the effects of sex [Fig. 3 *A* and *B*; *Grin2d*, F_(1,16)_ = 1.25, *P *> 0.05; *Grm4,* F_(1,16)_ = 0.34, *P* > 0.05] nor the interaction between drinking water treatment and sex [*Grin2d*; F_(1,16)_ = 0.45, *P* > 0.05; *Grm4*, F_(1,16)_ = 0.01, *P *> 0.05] were significant. Thus, aspartame exposure up-regulated *Grm4* and *Grin2d* mRNAs in male and female amygdala. The expression of *Gabarap* mRNA showed a significant effect of drinking water treatment [Fig. 3*C*; aspartame < plain drinking water; two-way ANOVA; F_(1,16)_ = 15.27, *P* < 0.01], but the effects of sex [Fig. 3*C*; F_(1,16)_ = 0.12, *P* > 0.05] and the interaction between drinking water treatment and sex [F_(1,16)_ = 0.02, *P *> 0.05] were not significant. Thus, the aspartame exposure down-regulated *Gabarap* mRNA expression in male and female amygdala.

In summary, RNA sequencing and quantitative PCR revealed significant changes in the glutamatergic and GABAergic synapse pathway in the amygdala in male mice exposed to 0.03% aspartame. These data together with the diazepam data suggest a shift in the excitation-inhibition (or glutamate-GABA signaling, respectively) balance in the amygdala toward excitation (or glutamatergic signaling).

### Transgenerational Transmission.

Recent evidence suggests that a variety of environmental factors produce phenotypes that are inherited by multiple generations descending from the directly exposed individuals (55–66). Whether the anxiety produced by aspartame exposure shows heritability was not known. To address this issue, we bred male mice exposed to 0.03% or 0.015% aspartame-containing drinking water for 12 wk with aspartame-naïve female mice to produce the F1 generation. The F0 males were being exposed to aspartame at the time of the breeding. A parallel set of male and female mice consuming plain drinking water were bred to produce another set of F1 mice. Thus, three paternal lineages of F1 mice were produced: the 0.03% and 0.015% aspartame lineages and the plain drinking water lineage. We did not generate F1 mice from female aspartame—or plain drinking water exposed founders. In other words, we did not examine maternal lineages.

We produced the F1 mice from F0 male mice following 12 wk of aspartame exposure, even though anxiety was evident in the F0 mice following only 6 wk of aspartame exposure (Fig. 1*A*) because our previous data showed that 12 wk of exposure to saccharin (56), another artificial sweetener or nicotine (67) was required to produce heritable phenotypes.

The F1 male mice derived from the 12-wk paternal 0.03% aspartame lineage were bred with aspartame-naïve females to produce the F2 generation. A set of F1 male mice from F0 plain drinking water lineage were also bred with aspartame-naïve females to produce the F2 generation. Thus, two paternal lineages of F2 mice were derived: F1 0.03% aspartame lineage and F1 plain drinking water lineage. We did not produce F2 mice from the 0.015% aspartame lineage.

In the present study, our focus is on patrilineal transmission. Therefore, we did not examine maternal lineages, although matrilineal transmission of the effects of environmental factors has been reported [Review in ref. 68].

The potential effects of aspartame lineage on developmental milestones in the early postnatal period were examined in the F1 and F2 generations. The litter size at birth; body weight at each of the first three postnatal weeks; time of eye opening, ear detachment, or fur appearance did not show significant effects of paternal lineage in F1 or F2 generations (*SI Appendix*, Table S5).

**Fig. 4.**
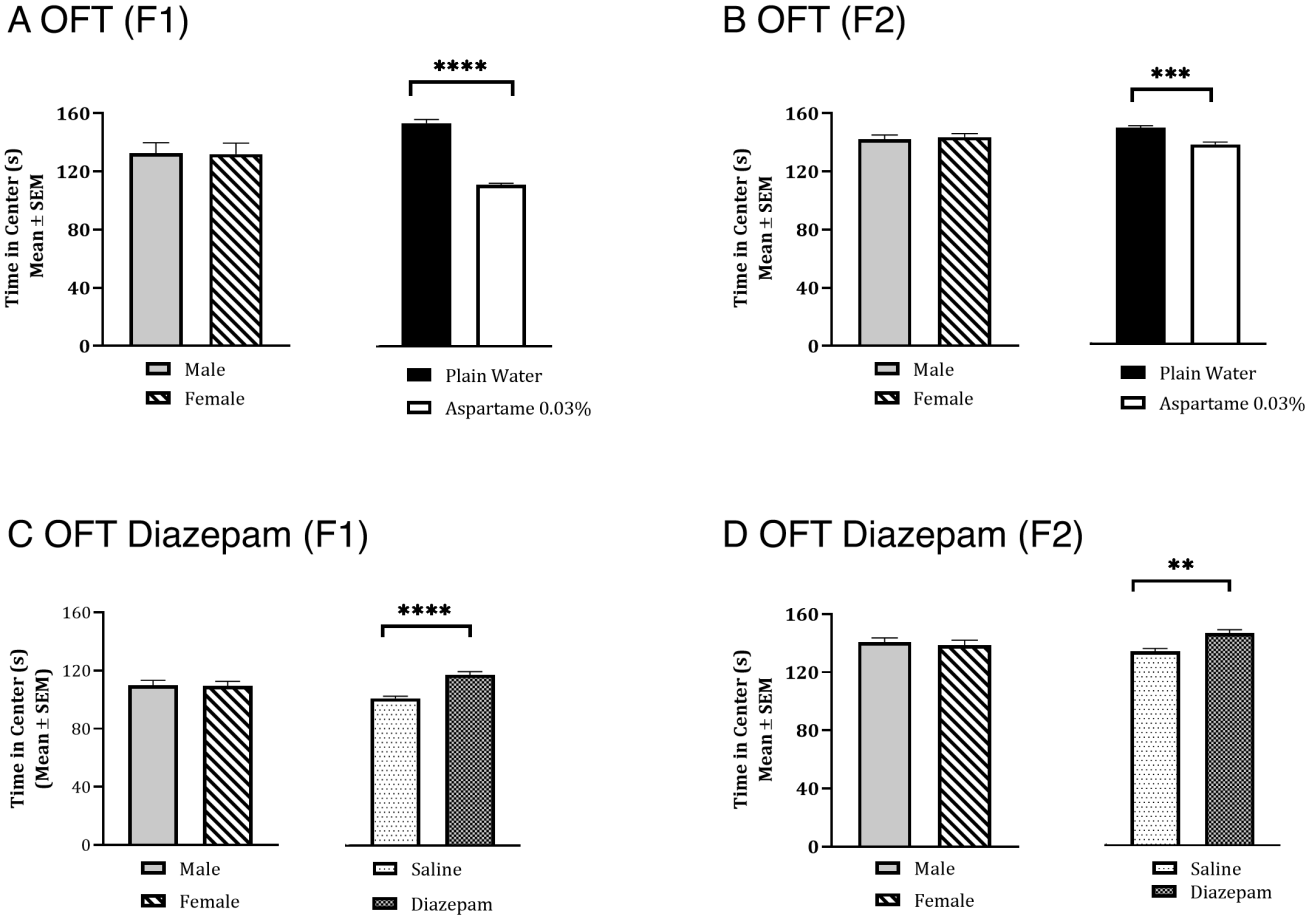
Anxiety-like behavior in male and female mice in the F1 (*A*) and F2 (*B*) generations descending from the paternal aspartame lineage and response to diazepam (*C* and *D*) were analyzed using the open field test (OFT). Two-way ANOVA showed no significant effect of sex in F1 or F2 generations. Therefore, data from male and female mice were analyzed together. The mice from the paternal aspartame lineage spent significantly shorter time in the center areas of the open field compared to their counterparts from the plain water lineage in F1 (*A*) and F2 (*B*) generations (male + female combined). Male and female mice from F1 and F2 generations were examined in the OFT 30 min following a single intraperitoneal administration of saline (*C* and *D*). The same mice received diazepam (3 mg/kg, i.p.) 48 h. later and were re-examined in the OFT 30 min after the diazepam administration. Repeated Measures ANOVA did not show significant effect of sex. Therefore, data from male and female mice were combined and analyzed. The time spent in the center areas was significantly reduced following the diazepam administration compared to the earlier saline administration in F1 (*C*) and F2 (*D*) generations (male + female). *** = *P *< 0.001; **** = *P *< 0.0001.

The time spent in the center areas in the OFT in F1 and F2 mice from the 0.03% aspartame or plain water lineages showed a significant effect of paternal lineage [Fig. 4 *A* and *B*; aspartame < plain drinking water, two-way ANOVA; F1: F_(1,16)_ = 222.1; *P* < 0.0001; F2: F_(1,12)_ = 24.03, *P *< 0.001], but the effects of sex [F1: F_(1,16)_ = 0.057; *P* > 0.05; F2: F_(1,12)_ = 0.38, *P *> 0.05] or the interactions between lineage and sex [F1: F_(1,16)_ = 0.12; *P* >0.05; F2: F_(1,12)_ = 0.79, *P *> 0.05] were not significant. The total distance traveled in the OFT did not show significant effects of paternal lineage [*SI Appendix*, Fig. S2*A*: two-way ANOVA; F1: F_(1,16)_ = 0.03, *P *> 0.05; F2: F_(1,12)_ = 0.08, *P *> 0.05], sex [F1: F_(1,16)_ = 0.0006, *P *> 0.05; F2: F_(1,12)_ = 0.003, *P *> 0.05], or interaction between lineage and sex [F1: F_(1,16)_ = 0.1, *P *> 0.05; F2: F_(1,12)_ = 0.01, *P *> 0.05].

We performed OFT in F1 mice derived from the 0.015% aspartame or plain water lineages. The time spent in the center areas in the OFT showed a significant effect of paternal lineage [*SI Appendix*, Fig. S2*C*; two-way ANOVA F_(1,16)_ = 59.47, *P *< 0.0001], but the effects of sex [*SI Appendix*, Fig. S2*C*; F_(1,16)_ = 1.86, *P *> 0.05] and the interaction between lineage and sex [F_(1,16)_ = 2.19, *P *> 0.05] were not significant. The total distance traveled in the OFT did not show significant effects of paternal lineage [*SI Appendix*, Fig. S2*D*; two-way ANOVA; F_(1,16)_ = 0.05, *P *> 0.05], sex [F_(1,16)_ = 1.05, *P *> 0.05] or interaction between lineage and sex [F_(1,16)_ = 1.52, *P *> 0.05].

In summary, male and female mice in the F1 generation derived from the 0.03% and 0.015% paternal aspartame lineage showed significant anxiety-like behavior, suggesting that anxiety produced by both doses of aspartame was heritable. The F2 male and female mice from the 0.03% paternal lineage also showed anxiety, demonstrating transmission of aspartame-induced anxiety up to two generations.

Next, we examined whether the anxiety in the F1 and F2 generations produced from the 0.03% aspartame paternal lineage responded to diazepam treatment in a manner consistent with that of the F0 founder mice. We analyzed the time spent in the center areas in the OFT in F1 and F2 mice 30 min following a single diazepam (3 mg/kg; i.p.) or vehicle administration. There was a significant effect of diazepam [Fig. 4 *C* and *D*; diazepam > saline two-way Repeated Measures ANOVA; F1: F_(1,16)_ = 38.85, *P *< 0.0001; F2: F_(1,12)_ = 15.6, *P *< 0.01], but the effects of sex [Fig. 4 *C* and *D*; F1: F_(1,16)_ = 0.03, *P *> 0.05; F2: F_(1,12)_ = 0.40, *P *> 0.05] or interactions between sex and diazepam treatment [F1: F_(1,16) _= 0.71, *P *> 0.05; F2: F_(1,12)_ = 0.001, *P *> 0.05] were not significant. The total distance traveled in the OFT did not show significant effects of diazepam [*SI Appendix*, Fig. S4 *B* and *C*; two-way ANOVA; F1: F_(1,16)_ = 0.03, *P *> 0.05; F2: F_(1,12)_ = 0.08, *P *> 0.05], sex [F1: F_(1,16)_ = 0.001, *P *> 0.05; F2: F_(1,12)_ = 0.003, *P *> 0.05], or interaction between sex and diazepam [F1: F_(1,16)_ = 0.01, *P *> 0.05; F2: F_(1,12)_ = 0.01, *P *> 0.05].

Thus, the anxiety was significantly reduced by diazepam in the F1 and F2 generations suggesting that the changes in glutamate-GABA signaling attributed as a mechanism for the anxiety behavior in the F0 generation may be the mechanism in the F1 and F2 generations as well. In other words, not only the behavioral phenotype but also the mechanistic basis of the phenotype showed transgenerational transmission from F0 to F1 to F2 generation.

Although diazepam administration increased the time spent in the center areas in the OFT in male and female aspartame-exposed mice in F0, F1, and F2 generations, we examined whether the diazepam treatment “normalized” the behavior in the aspartame group in all three generations compared to the behavior of male and female mice in the plain drinking water group receiving saline (i.e., at baseline). For this analysis, we considered the F0 male and female mice in the plain drinking water group that received saline as the single baseline control group (rather than using separate F0, F1, and F2 plain water + saline controls), because there was no significant difference in time spent in the center areas among males and females in the three groups [one-way ANOVA, F_(2,23)_ = 2.42, *P *> 0.05].

Two-way ANOVA revealed that the diazepam treatment produced significant effects [F_(3,26)_ = 29.61, *P *< 0.0001], but neither the effect of sex [F_(1,26)_ = 0.56, *P *> 0.05] nor that of the interaction between sex and diazepam [F_(3,26)_ = 0.58, *P *> 0.05] was significant. Dunnett’s multiple comparisons test (*SI Appendix*, Fig. S4*D*) showed significant differences in the time spent in the center areas in F0 and F1 mice between aspartame group receiving diazepam and the plain drinking water group receiving saline vehicle [plain drinking water + saline > aspartame + diazepam; male = *P* < 0.01; females = *P *< 0.001]. However, the same comparisons for the F2 generation did not show significant differences [*SI Appendix*, Fig. S4*D*; *P *> 0.05].

To examine whether the changes in amygdala gene expression also showed transgenerational transmission, we used quantitative PCR to analyze mRNA expression of *Grm4, Grin2d,* and *Gabarap* in the amygdala of male and female F1 and F2 mice. The mRNA for all three genes in the F1 generation showed a significant effect of lineage [Fig. 3 *D*–*F*; two-way ANOVA; aspartame > plain drinking water*: Grin2d*, F_(1,8)_ = 6.63, *P *< 0.05; *Grm4* F_(1,8)_ = 6.98, *P *< 0.05; aspartame < plain drinking water: *Gabarap*, F_(1,16)_ = 14.23, *P *< 0.01], but neither the effect of sex [*Grin2d*, F_(1,8)_ = 0.01, *P *> 0.05; *Grm4,* F_(1,8)_ = 1.40, *P *> 0.05; *Gabarap*, F_(1,16)_ = 0.06, *P *> 0.05] nor the interaction between lineage and sex [*Grin2d*, F_(1,8)_ = 0.31, *P* > 0.05; *Grm4,* F_(1,8)_ = 0.01, *P *> 0.05, *Gabarap*, F_(1,16)_ = 0.08, *P *> 0.05] was significant.

However, mRNA expression for none of the three genes in the F2 generation showed significant effects of lineage [*SI Appendix*, Fig. S3 *A*–*C*; two-way ANOVA; *Grin2d*, F_(1,8)_ = 2.93, *P *> 0.05; *Grm4*, F_(1,8)_ = 2.09, *P *> 0.05; *Gabarap*, F_(1,12)_ = 0.89, *P *> 0.05], sex [*Grin2d*, F_(1,8)_ = 0.38, *P *> 0.05; *Grm4*, F_(1,8)_ = 0.1, *P *> 0.05; *Gabarap*, F_(1,12)_ = 0.14, *P *> 0.05], or interaction between lineage and sex [*Grin2d*, F_(1,8)_ = 0.01, *P *> 0.05; *Grm4*, F_(1,8)_ = 0.14, *P *> 0.05; *Gabarap*, F_(1,12)_ = 0.003, *P *> 0.05].

Thus, the mRNA expression data showed that the aspartame-induced changes in *Grin2d*, *Grm4,* and *Gabarap* were transmitted from the F0 to F1 generation but not from F1 to F2 generations.

## Discussion

Our data show that daily consumption of aspartame via drinking water at doses that are the mouse equivalents of 7 to 15% of the human maximum DIV or 2–4 small (8 oz) cans of aspartame-sweetened diet soda produces anxiety in male and female mice. The anxiety is alleviated by diazepam, a positive allosteric modulator of the GABA-A receptor and a drug used in the treatment of generalized anxiety disorder. The aspartame exposure disrupts gene expression in the amygdala, a brain region associated with regulation of anxiety and fear responses. Perhaps more importantly, especially from a public health perspective, the aspartame-induced anxiety, its response to diazepam, and the changes in amygdala gene expression are heritable along the paternal lineage.

Since the aspartame-induced anxiety was alleviated by a single administration of diazepam, downregulation of GABA-A receptor signaling appears to be an underlying mechanism. RNA sequencing and quantitative PCR demonstrated downregulation of three GABA-A receptor associated genes, namely *Gabarap*, *Gabra3,* and *Gabrg2* in the amygdala. In addition, genes for three G_i/o_ protein subunits, namely *Gng13*, *Gng4,* and *Gng10* that are associated with GABA-B autoreceptor signaling were also down-regulated, suggesting that aspartame’s effects in the amygdala on GABA neurotransmission may include the ionotropic GABA-A receptor as well as the metabotropic GABA-B receptor.

The RNA sequencing and quantitative PCR data showed that aspartame exposure also influenced glutamatergic signaling in the amygdala. The expression of genes for NMDA receptor (*Grin2d*), metabotropic receptor (*Grm4*), and kainite receptor (*Grik5*) were up-regulated. The downregulation of the presynaptic G_i/o_ protein subunit genes *Gng13*, *Gng4* and *Gng10* could contribute to increased synaptic release of glutamate, because G_i/o_ proteins serve as second messengers for metabotropic glutamate receptor signaling, which exerts negative feedback on glutamate release from the presynaptic terminal. Therefore, downregulation of the presynaptic G_i/o_ proteins could augment glutamate release at the synapse. Thus, the overall effect of aspartame at the glutamatergic synapse may occur via upregulation of postsynaptic NMDA, metabotropic and kainite receptor expression, and downregulation of presynaptic metabotropic autoreceptor signaling. Collectively these effects would enhance glutamatergic signaling in the amygdala.

A third possibility of aspartame-induced enhancement of excitatory tone in the amygdala is via aspartic acid, a metabolite of aspartame and an excitatory neurotransmitter, which increases synaptic glutamate levels and NMDA receptor expression and activity (7). In addition, aspartame exposure up-regulated *Glul* (glutamate-ammonia ligase), a key enzyme in the biosynthesis of glutamate and GABA as well as *Cacna1A*, *Cacna1C,* and *Cacna1D* (calcium voltage-gated channel subunit genes) that regulate voltage-gated calcium channel signaling.

In summary, our RNA sequencing and quantitative PCR data offer compelling evidence for upregulation of excitatory glutamatergic signaling and downregulation of inhibitory GABAergic signaling in the amygdala.

The dopaminergic synapse KEGG pathway was at the #11 position of enriched pathways in the amygdala of aspartame-exposed male mice (Fig. 2*A*), suggesting that aspartame’s effects may extend beyond the glutamate-GABA axis and involve dopaminergic signaling in the amygdala. Aspartame’s effects on dopamine could occur as a result of increases in plasma and brain concentrations of phenylalanine (3–5, 11, 69–71), another metabolite of aspartame. However, since our focus was on the glutamatergic and GABAergic neurotransmission, we did not pursue further the changes in dopaminergic synapse pathway.

The presence of anxiety, its response to diazepam, and the changes in amygdala gene expression in the F1 generation suggest intergenerational (i.e., from the aspartame-exposed father to his “children”) heritability of aspartame’s effects, likely due to aspartame-induced changes in the F0 spermatozoa. The anxiety and its response to diazepam in the F2 generation suggest transgenerational transmission (i.e., from the aspartame-exposed male to his “grandchildren”) (63, 68), which would have occurred if the F1 spermatozoa that produced the F2 generation had “inherited” changes from the F0 generation.

The changes in the spermatozoa that can produce heritability of phenotypes may be genetic (e.g., mutations) or epigenetic (i.e., chemical modification of DNA/histones or expression of non-coding RNA). Environment-induced epigenetic changes typically are transient and even reversible over time, whereas changes produced by genetic mutations are generally stable across successive generations (58, 72–76). In the present study, aspartame-induced changes showed progressive attenuation during F0 to F1 to F2 descent. For example, quantitative PCR showed significant changes in glutamatergic and GABAergic gene expression in the F0 and F1 generations, but the changes were no longer significant in the F2 generation. Similarly, the OFT data showed a decline in the magnitude of anxiety (i.e., time spent in the center areas) during descent from F0/F1 (plain water versus aspartame difference was approximately 30%) to F2 (only 8% difference) generations. In addition, the diazepam administration reduced the anxiety-like behavior to baseline in the F2 generation, but not in the F1 and F2 generations. One interpretation of these observations is that the aspartame-induced anxiety-like behavior attenuated gradually during descent from F0 to F2. Finally, every offspring in every generation in the aspartame lineage showed anxiety and response to diazepam, suggesting non-Mendelian heritability. Thus, our findings favor aspartame-induced epigenetic changes in the spermatozoa as a likely mechanism of heritability of the phenotypes.

The experimental design pursued in previous studies of aspartame’s effects in preclinical models has varied significantly [dose, route, frequency, and duration of aspartame exposure; (7, 8, 13–23)]. In these studies, generally, the period of aspartame exposure was 30 d or shorter. We found that aspartame exposure for 6–8 wk was necessary for the anxiety-like behavior to emerge. In fact, one study that administered aspartame in drinking water for 28 d in a rat model reported anxiety-like behavior (19). Therefore, we believe that differences in experimental design are among the factors that may have contributed to inconsistency between our findings and previous findings on aspartame’s effects on anxiety-like behavior.

In conclusion, our findings offer unequivocal evidence for aspartame-induced anxiety in male and female mice. Downregulation of GABA signaling and upregulation of glutamate signaling in the amygdala contribute to the anxiety. The anxiety and the underlying changes in glutamate-GABA signaling in the amygdala are heritable along the paternal line of descent. These data do not merely add anxiety to the long list of aspartame’s adverse effects, but also raise the previously unrecognized possibility that aspartame’s adverse effects are not limited to the individuals who consume it but persist for generations to come. Therefore, aspartame deserves a place on the list of environmental agents such as hormones, insecticides, and drugs of abuse whose adverse effects are not limited to the exposed individuals but manifest in multiple generations of descendants (55–66, 77).

## Methods

### Animals.

C57BL/6 mice (Charles River Laboratories, Wilmington, MA) were maintained in the Florida State University Laboratory Animal Resources facility in a humidity- and temperature-controlled room on a 12-h light-dark cycle with ad libitum access to food and water. The mice of the same sex were pair-housed except during breeding, pregnancy, and nursing. All the procedures described below were approved by the institutional animal care and use committee and were carried out during the lights-off period under a red light. Experimental procedures and analyses were performed by investigators “blinded” to the experimental group assignment of the mouse and/or through automated scoring procedures to avoid potential experimenter bias.

### Aspartame Delivery via Drinking Water.

Male and female mice (8-10-wk-old) were randomly assigned to receive plain drinking water or drinking water containing 0.03%, 0.015%, or 0.0075% aspartame (Sigma Aldrich, # A5139) for up to 18 wk, with a fresh supply of each type of water provided weekly. Body weights of the mice were measured at weekly intervals.

### Aspartame Preference Using a Two-Bottle Choice Test.

Male and female mice were given free access to two water bottles, one containing plain drinking water and the other containing either 0.03% or 0.015% aspartame-containing water. We measured weekly water consumption over a 12-wk period in male mice and a 6-wk period in female mice.

### Behavioral Analyses.

At 2-wk intervals beginning at 4 wk of the 12-wk period of exposure to 0.03% aspartame, 0.015% aspartame or plain drinking water, anxiety-like behavior was examined using the open field test (OFT; n = 8/sex/group). The OFT consists of placing the mouse at the center of an open-top box (40 cm^3^) and allowing the mouse to explore the box for 10 min. The activity of the mouse is recorded using an overhead video camera. At the end of the 10-min period, the time spent in the center versus the periphery of the box as well as the total distance traveled was analyzed using a video tracking software (AnyMaze, Wood Dale, IL).

Another set of mice was tested on the elevated zero maze (EZM) (n = 8/sex/group) at the end of the 12-wk drinking water exposure period. The EZM is a circular, elevated maze consisting of continuous alternating open and closed areas arranged in a circle (Maze Engineers, Boston, MA). The mouse was placed in an open area of the EZM and allowed to explore the maze freely for 5 min. Video recordings were evaluated by eye by an investigator blinded to the identity of the mouse. The time spent in open and closed areas and the number of entries into each area were recorded.

### Production of Multiple Generations.

Male mice from the 0.03% and 0.015% aspartame groups were bred with aspartame-naïve female mice following 12 wk of aspartame exposure to produce the F1 generation. A set of male and female mice consuming plain drinking water were bred together to produce the F1 generation. Thus, three paternal lineages of F1 mice were produced: the 0.03% and 0.015% aspartame lineages and the plain drinking water lineage. All eight male mice in each of the two aspartame groups and the plain water group were bred with females, which produced five litters each from the plain water and 0.03% aspartame group, and four litters from the 0.015% aspartame group. From each litter, not more than two males and two females were used in the experimental analyses.

In an initial study, we also produced F1 litters from the three groups of male mice following 8 wk of drinking water exposures. However, these litters did not show significant effects of paternal lineage in the OFT and were therefore excluded from further analysis.

The F1 male mice derived from the 12-wk paternal 0.03% aspartame lineage were bred with aspartame-naïve females to produce the F2 generation. A set of F1 male mice from F0 plain drinking water lineage were also bred with aspartame-naïve females to produce the F2 generation. Thus, two paternal lineages of F2 mice were derived: F1 0.03% aspartame lineage and F1 plain drinking water lineage. Once again, all eight male mice in each of the two F1 groups were bred with females. However, only four litters per group were produced and were used in the study. From each litter, not more than two males and two females were used in the experimental analyses.

We did not produce F2 mice from the 0.015% F1 aspartame lineage. Similarly, maternal lineages (neither 0.03% nor 0.015% exposure) were not produced from any group.

### Behavioral Analysis in F1 and F2 Generations.

The F1 and F2 male and female mice from each paternal lineage were examined in the OFT at approximately 65–70 d of age.

### Analysis of Response to Diazepam.

Diazepam (SKU: 054902, Covetrus, Ocala, FL) or saline (vehicle) was administered intraperitoneally to aspartame-naïve male and female mice. In a pilot study, male and female mice from the plain drinking water group (n = 4–6) were administered saline, and 30 min later, distance traveled in the OFT was analyzed. The data were used as baseline data. 2 d later, the same mice received a single i.p. administration of diazepam at 2, 3, or 4 mg/kg dose. The distance traveled in the OFT was analyzed 30 min after the diazepam administration. The maximum dose of diazepam that did not produce significant reductions in the total distance traveled (a proxy for sedative effects) was established to be 3 mg/kg, and it was chosen as the dose in the next set of analyses. Next, male and female mice from the F0, F1, and F2 0.03% aspartame lineages were administered saline (n = 8 (F0) and 4 (F1 and F2) per group, volume same as that of the diazepam solution) and 30 min later subjected to OFT to establish baseline measures for time spent in the center areas of the OFT. 2 d later, the same mice received diazepam (3 mg/kg; i.p.), and 30 min later, the time spent in the center areas and the total distance traveled in the OFT were analyzed and compared to these measures at baseline (i.e., following the saline administration).

### Tissue Collection.

Male and female mice from F0, F1, and F2 generations were anesthetized (Isoflurane, Pivetal, Liberty, MO) decapitated, and the brains were dissected upon completion of behavioral analysis. Amygdala was collected by microdissection and snap-frozen in liquid nitrogen. Another set of male mice from the F0 plain water and 0.03% aspartame and 0.015% aspartame groups (n = 6 (F0) and 4 (F1 and F2) per group) were used for analysis of serum metabolic biomarkers following 18 wk of drinking water exposures. The mice were anesthetized with Isoflurane, decapitated, and their trunk blood was collected. Serum samples were used for analysis of metabolic biomarkers by ELISA (IDEXX Laboratories, North Grafton, MA).

### RNA Sequencing.

Following completion of the behavioral analyses, the amygdala samples from the male F0 mice exposed to 0.03% aspartame or plain drinking water (n = 4 per group) for 12 wk were processed for RNA extraction (Qiagen; RNeasy mini kit). Female mice were not used in this analysis. A next generation sequencing library was prepared for each sample using the Illumina TruSeq stranded mRNA library kit. Libraries were barcoded for multiplexing with IDT for Illumina unique dual indexes. Each multiplexed library was sequenced on an Illumina NovaSeq 6000 as a 150 base pair paired-end sequencing run. Quality control analysis of each library was performed using fastQC (http://www.bioinformatics.babraham.ac.uk/projects/fastqc/). Adapter trimming was performed as part of individual library demultiplexing. Illumina RNA-Seq Alignment (Version 2.0.2) was used, including STAR Aligner (78) to align and map sequencing reads to the mouse genome (genome release UCSC mm10). Read counts for each gene were generated by Salmon (79) and used as a measure of abundance for differential gene expression analysis. DESeq2 (80) was used to generate a Principle Component Analysis (PCA) plot and to determine statistically significant DEGs (a False Discovery Rate, FDR, of <0.05 was used). Results of the PCA analysis required exclusion of one sample from the aspartame group from further analysis, based on criteria established previously (81). The analysis yielded a list of mRNAs present at significantly different levels between the aspartame and plain drinking water groups and provided a measure of confidence of each difference. Genes with a statistically significant differential expression were further analyzed by Kyoto Encyclopedia of Genes and Genomes (KEGG) pathway analysis using Webgestalt (82, 83) to establish possible functional roles (84–87). All data from these analyses are available in the NCBI Gene Expression Omnibus https://www.ncbi.nlm.nih.gov/geo/query/acc.cgi?acc=GSE210561 (Accession # accession GSE210561).

### Quantitative PCR.

Amygdala tissue samples from both male and female mice (n = 6/sex/lineage) were processed for quantitative PCR. Reverse transcription reactions were performed using the SuperScript III cDNA synthesis kit (Invitrogen, Waltham, MA; Cat#:18080-051). Primer sequences for the following genes were used based on previously published data: RNA 18s ribosomal 1 (*Rna18s1*; Invitrogen, Cat#: Hs Hs99999901_s1) (67), NMDA receptor 2D (*Grin2d*; Invitrogen, Cat#: Mm00433822_m1) (88), metabotropic receptor 4 (*Grm4*; Invitrogen, Cat#: Mm01306128_m1) (89), and GABA-A receptor associated protein (*Gabarap*; Invitrogen, Cat#: Mm00490678_m1) (90). Quantitative real time-PCR was performed in a StepOne Plus Thermocycler (Life technologies) using Taqman Fast Advanced PCR Master Mix (Invitrogen; Cat#: 4444557) through 40 PCR cycles (95°C for 20 s, 95°C for 1 s, 60°C for 20 s). Expression of each mRNA was normalized to *Rna18s1* expression.

### Statistical Analyses.

For analyses of the data from the F0 generation, the number of male and female mice in each experimental group was considered as the “n” for that group. For the analyses of data from the F1 and F2 generations, the number of litters contributing offspring to a given experimental group was used as the “n” for that group. Statistical significance of drinking water preference in the two-bottle paradigm was tested using a one-sample *t* test by considering 50 as the theoretical mean had no preference existed (i.e., 50% consumption of each of the two types of drinking water). A Repeated Measures two-way ANOVA followed by Dunnett’s multiple comparisons tests was used for the OFT analysis of F0 mice at 2-wk intervals. For the diazepam OFT data, a Repeated Measures two-way ANOVA was used. For all the other analyses involving more than one variable, two- or three-way ANOVA was used to test statistical significance of main effects and interactions. The statistical significance of the effects of a single variable was analyzed using one-way ANOVA or unpaired *t* test. The analyses were performed using Prism 9.2.0 software (GraphPad Prism, San Diego, CA).

## Supplementary Material

Appendix 01 (PDF)

## Data Availability

All RNA sequencing data are available in the NCBI Gene Expression Omnibus https://www.ncbi.nlm.nih.gov/geo/query/acc.cgi?acc=GSE210561 (Accession # accession GSE210561). All study data are included in the article and/or *SI Appendix*.
