# Supplementary material for: Transgenerational transmission of aspartame-induced anxiety and changes in glutamate-GABA signaling and gene expression in the amygdala
Source: Proc Natl Acad Sci U S A. 2022 Dec 2;119(49):e2213120119. doi: 10.1073/pnas.2213120119 (PMC9894161; doi:10.1073/pnas.2213120119)
Supplement: Supplementary file 1 — Appendix 01 (PDF) [file pnas.2213120119.sapp.pdf]

Supporting Information

for

Transgenerational transmission of aspartame induced anxiety, and changes in glutamate-GABA signaling and gene expression in the amygdala

Sara K. Jones[1], Deirdre M. McCarthy[1], Cynthia Vied[2], Gregg D. Stanwood[1], Chris Schatschneider[3] and Pradeep G. Bhide[1*]

[1]Biomedical Sciences, [2]Translational Science Laboratory and [3]Psychology, Florida State University, Tallahassee, FL 32306

Correspondence to: Pradeep G. Bhide, Ph.D., Biomedical Sciences, FSU College of Medicine, 1115, West Call Street, Tallahassee, FL 32306; Telephone: 850-645-9847

Email: Pradeep.bhide@med.fsu.edu

Major – Biological Sciences; Minor: Neuroscience

Key words: Artificial sweetener, Emotional behavior, Intergenerational transmission, GABA

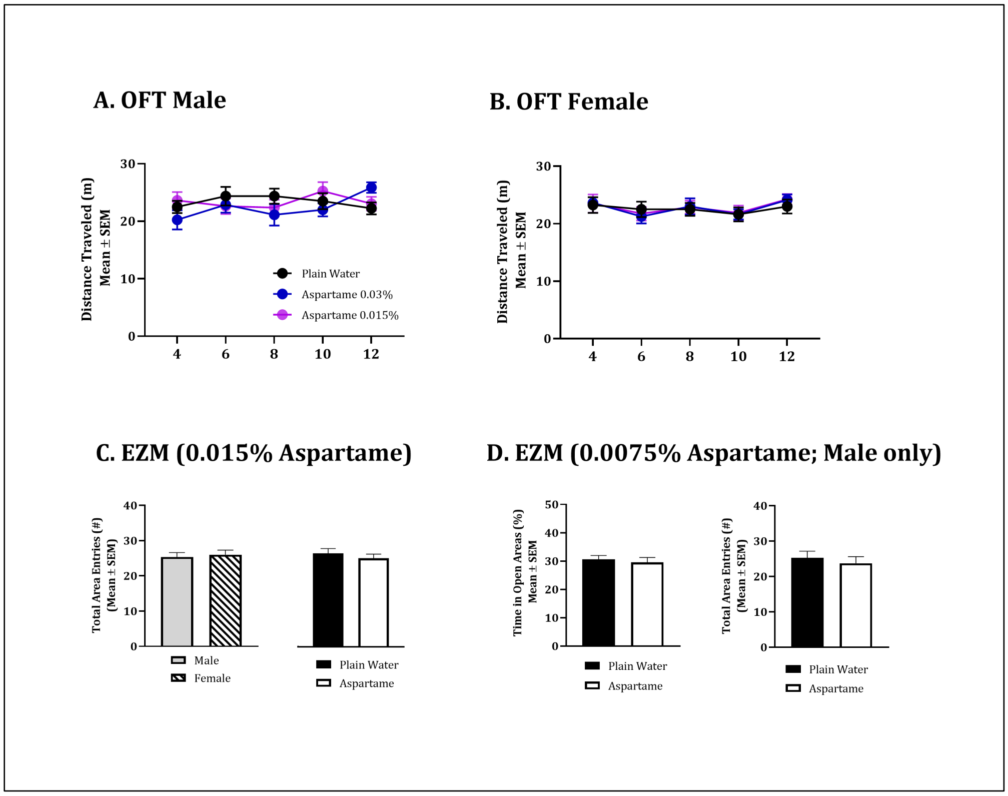

Figure S1: Analysis of exploratory behavior and locomotion as well as anxiety in male and female mice exposed to plain drinking water or aspartame in the open field test (OFT; A, B) and elevated zero maze (EZM; C, D). Two-way ANOVA of the total distance traveled (a measure of exploration and locomotion) in the OFT did not show significant effects of 0.03% (blue) or 0.015% (purple) aspartame exposure compared to plain drinking water (black) in male (A) or female (B) mice. Two-way ANOVA of the total number of arm entries in the EZM (a measure of exploration and locomotion) did not show significant effect of sex or 0.015% aspartame (C). Exposure to 0.0075% aspartame did not produce significant effects on the time spent in the open areas of the EZM (a measure of anxiety; D) or the total number of arm entries (D) in male mice. Female mice in the 0.075% aspartame group were not examined.

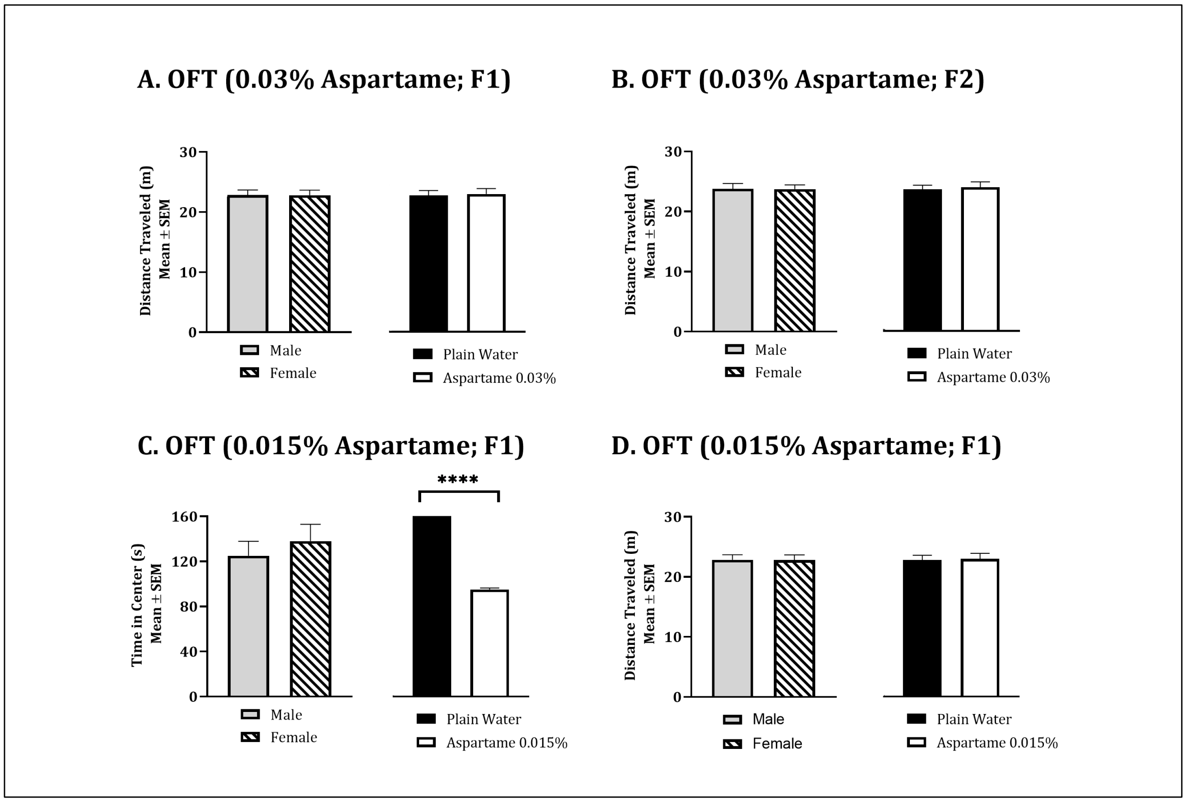

Figure S2: Analysis of the total distance traveled (a measure of exploration and locomotion; A, B) and time spent in the center areas (a measure of anxiety; C, D) in the open field test (OFT) for F1 and F2 generations derived from 0.03% and 0.015% aspartame or plain drinking water paternal lineages. Two-way ANOVA did not show significant effects of sex for F1 (A) or F2 (B) generations from the 0.03% paternal aspartame lineages. Therefore, data from male and female mice were combined. There was no significant effect of lineage for the total distance traveled in F1 (A) or F2 (B) generations from the 0.03% aspartame group. Two-way ANOVA of the 0.015% data did not show significant effects of sex for the time in the center (C) or total distance traveled (D) in F1 generation derived from the 0.015% aspartame lineage. Therefore, data from male and female mice were combined. There was a significant effect of paternal lineage (0.015% aspartame versus plain water) in the time spent in the center areas (C) but not on the total distance traveled in the F1 generation (D). **** = $p<0.0001$.

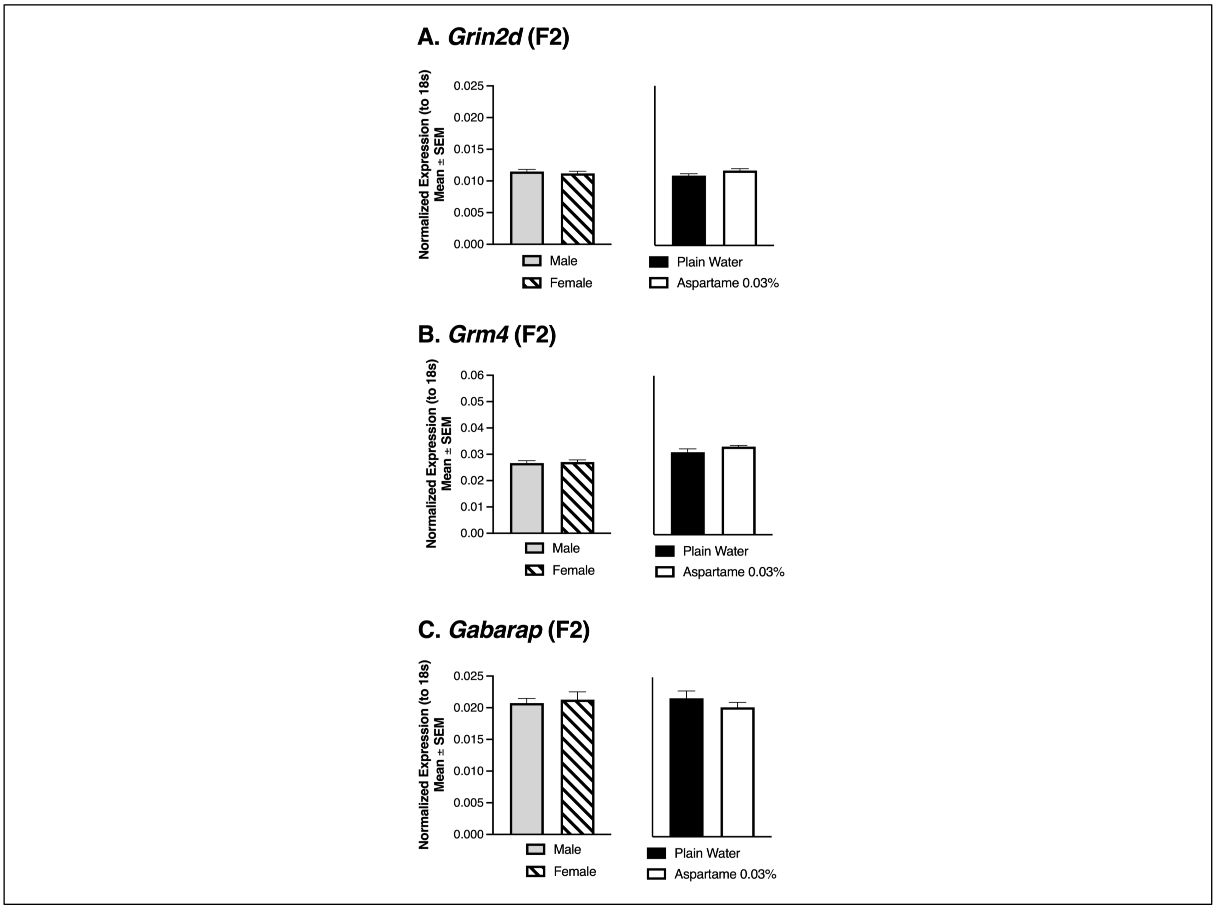

Figure S3: Quantitative PCR analysis of expression of mRNAs for *Grin2d* (A), *Grm4* (B) and *Gabarap* (C) in male and female mice from the F2 generation derived from aspartame or plain water F1 lineages. In each case, expression of the mRNA of interest was normalized to that of ribosomal 18s RNA. Two-way ANOVA did not show significant effects of sex or paternal lineage for any of the mRNA.

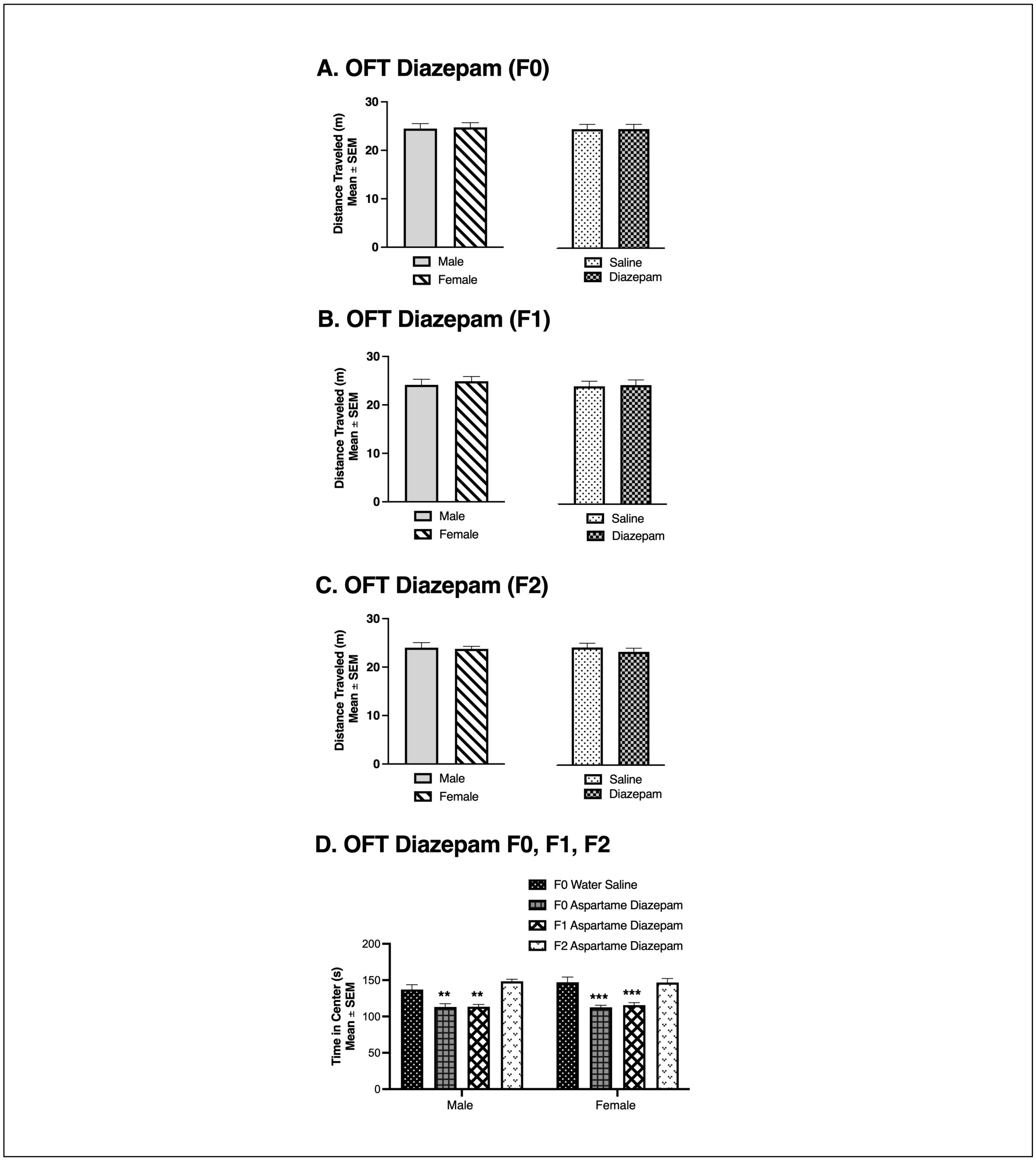

Figure S4: Analysis of the OFT data from aspartame-exposed mice in F1, F2 and F3 generations. The total distance traveled in the open field test (OFT) as a measure of exploratory behavior and locomotion in male and female mice 30 min after a single intraperitoneal administration of saline (at baseline) or diazepam (3 mg/kg, 48 hr. after the saline administration) in F0 (A), F1 (B) and F2 (C) generations. Repeated measures

ANOVA did not show significant effects of sex or diazepam treatment in any generation. The effects of diazepam on the time in the center were compared between the F0 male or female mice receiving saline (baseline) with F0, F1, and F2 aspartame exposed mice receiving aspartame (D). Two-way ANOVA showed significant effects of diazepam treatment but not sex or diazepam x sex interaction. Dunnett's multiple comparison test showed significant differences between F0 and F1 male and female aspartame groups receiving diazepam and the F0 plain water group receiving saline (baseline). However, there was no significant difference between F2 male and female aspartame groups receiving diazepam and the F0 plain water group receiving saline (baseline). ** = $p<0.01$; *** = $p<0.001$.

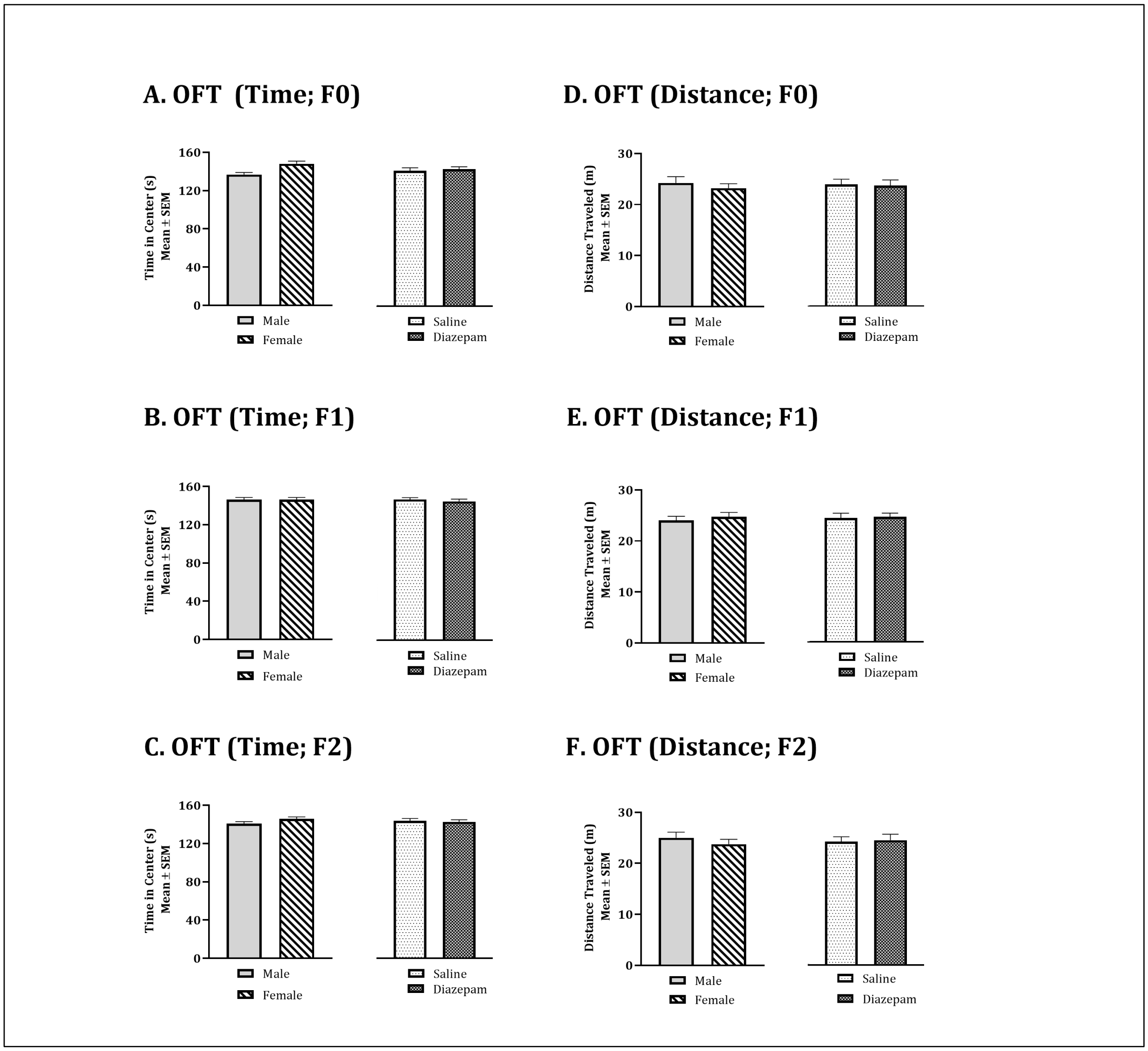

Figure S5: Data from the open field test (OFT) from F0, F1 and F2 generations from the plain water lineage. The time spent in the center areas (a measure of anxiety) and the total distance traveled (a measure of exploration and locomotion in male and female mice in the F0 (A, D), F1 (B, E) and F2 (C, F) generations was analyzed. The mice were tested in the open field 30 min after a single intraperitoneal administration of saline (at baseline) or aspartame (3 mg/kg, 48 hr. after the saline administration). Repeated measures ANOVA did not show significant effects of sex or diazepam in any generation for either measurement.

Table S1A

Water consumption (Mean±SEM) %			
Male		Female	
0.03% Aspartame	0.015% Aspartame	0.03% Aspartame	0.015% Aspartame
50.08±0.97	48.99±0.57	50.89±0.55	49.22±0.54

Table S1B

Experimental Groups	One-sample t-test			
	Male		Female	
	$t_{(df=7)}$	*p*	$t_{(df)}$	*p*
0.03% aspartame *versus* Plain water	0.08	0.93	1.62	0.15
0.015% aspartame *versus* Plain water	1.78	0.11	1.45	0.19

Table S1: Preference for drinking water containing 0.03% or 0.015% aspartame in a two-bottle choice paradigm by males over 12 weeks and by females over 6 weeks. (A) The volume of aspartame water consumed is expressed as a percentage of total water consumed (plain water + aspartame water = 100%). (B) Differences in consumption of aspartame-containing water and plain water were tested for statistical significance using a one-sample *t*-test, by considering 50 as the theoretical mean if no preference existed (i.e., if 50% of each type of drinking water would be consumed in the absence of a preference).

Table S2

Week #	Male [Mean±SEM (g)]			Female [Mean±SEM (g)]		
	Plain Water	0.03% Aspartame	0.015% Aspartame	Plain Water	0.03% Aspartame	0.015% Aspartame
1	20.65±0.39	20.91±0.30	20.82±0.20	20.13±0.60	20.88±0.21	19.81±0.50
2	22.06±0.38	21.65±0.31	21.70±0.23	21.08±0.66	21.68±0.23	20.59±0.49
3	23.58±0.49	22.59±0.46	23.38±0.39	21.82±0.65	22.69±0.23	21.50±0.51
4	24.53±0.58	23.15±0.46	24.42±0.49	22.52±0.73	23.39±0.26	22.29±0.50
5	25.19±0.51	23.95±0.51	25.40±0.6	23.29±0.68	24.22±0.24	23.13±0.49
6	25.60±0.60	24.19±0.51	26.112±0.72	24.24±0.70	25.11±0.25	24.08±0.52
7	26.25±0.62	25.36±0.51	27.02±0.67	*Not Analyzed*		
8	27.10±0.64	26.20±0.65	28.05±0.70			
9	27.73±0.74	26.59±0.66	28.80±0.75			
10	28.51±0.74	27.06±0.68	29.64±0.85			
11	29.45±0.69	27.99±0.75	30.15±0.85			
12	30.30±0.69	28.92±0.92	31.45±0.65			
**Mean±SEM**	**25.91±0.10**	**24.88±0.15**	**26.41±0.18**	**22.18±0.61**	**22.99±0.65**	**21.90±0.65**

Table S2: Weekly body weights of male and female mice consuming plain drinking water or drinking water containing 0.03% or 0.015% aspartame. The data were collected every week for 12 weeks for males and for 6 weeks for females.

Table S3

	Type of drinking water			One-way ANOVA	
**Analyte**	**Plain Drinking Water**	**0.015% Aspartame**	**0.03% Aspartame**	$F_{(2,21)}$	***p*-value**
Glucose (mg/dL)	202.5 ± 9.0	180.5 ± 4.1	194 ± 6.9	2.33	>0.05
Cholesterol (mg/dL)	103.8 ± 4.7	94.8 ± 6.4	100.7 ± 4.2	0.78	>0.05
Calcium (mg/dL)	8.9 ± 0.2	8.6 ± 0.1	8.7 ± 0.1	1.24	>0.05
Bicarbonate (mg/dL)	24.3 ± 1.4	26.0 ± 0.9	25.8 ± 0.5	0.81	>0.05
Aspartate Transferase (U/L)	262.0 ± 76.6	176.3 ± 29.8	281.2 ± 81.0	0.71	>0.05
Alkaline Phosphatase (U/L)	47.8 ± 2.0	39.7 ± 4.5	44.8 ± 0.8	2.05	>0.05
Alanine Transaminase (U/L)	37.0 ± 3.6	33.2 ± 5.0	41.5 ± 7.8	0.53	>0.05
Albumin (g/dL)	3.0 ± 0.1	2.6 ± 0.1	2.9 ± 0.1	2.62	>0.05
Total Bilirubin (mg/dL)	0.1 ± 0.0	0.1 ± 0.0	0.1 ± 0	1.15	>0.05
Globulin (g/dL)	2.3 ± 0.0	2.4 ± 0.1	2.4 ± 0.1	0.09	>0.05
Blood Urea Nitrogen (mg/dL)	30.8 ± 1.2	27.0 ± 1.2	30.7 ± 1.4	2.98	>0.05
Total Protein (g/dL)	5.3 ± 0.0	5.0 ± 0.1	5.3 ± 0.1	3.6	>0.05
Creatine kinase (U/L)	12796 ± 4681	7856 ± 2163	14322 ± 5473	0.6	>0.05

Table S3: Serum metabolic panel did not show significant changes in any one of the 13 biomarkers between male mice exposed to plain drinking water or drinking water containing 0.015% or 0.03% aspartame for 18 weeks.

Table S4A

Experimental groups	Distance Traveled (m) Mean±SEM	
	Male	Female
Diazepam 2 mg/kg	21.0 ± 1.4	20.8 ± 1.5
Diazepam 3 mg/kg	22.3 ± 1.3	21.2 ± 1.9
Diazepam 4 mg/kg	13.1 ± 1.5	13.0 ± 1.7
Saline	23.1 ± 1.4	22.5 ± 1.5

Table S4B

Comparisons between groups	Male		Female	
	t (df =18)	*p*	t (df =18)	*p*
Diazepam 2 mg/kg *versus* saline	1.28	>0.05	0.65	>0.05
Diazepam 3 mg/kg *versus* saline	0.9	>0.05	0.91	>0.05
Diazepam 4 mg/kg *versus* saline	4.3	<0.05	3.33	<0.05

Table S4: Diazepam dose-finding studies using total distance traveled in the OFT as a proxy for sedative effects. A single diazepam or saline administration via the intraperitoneal route was followed 30 min later by the OFT to analyze the total distance traveled by each of the three diazepam (2, 3 and 4 mg.kg) and saline groups (A). The maximum diazepam dose that did not produce significant reductions in the total distance traveled was 3 mg/kg (B), because 4 mg/kg dose produced significant reductions in the total distance traveled (B).

Table S5

Metric	F1 (Mean±SEM)					F2 (Mean±SEM)			
	**Plain Drinking Water**	**0.03% Aspartame**	**0.015% Aspartame**	**One-way ANOVA F(df)**	***p***	**Plain Drinking Water**	**0.03% Aspartame**	**Unpaired t-test $t_{(df)}$**	***p***
Litter Size	7.6 ± 0.50	6.8 ± 0.40	7.2 ± 0.50	0.75	>0.05	7.4 ± 0.50	7.0 ± 0.50	0.53	>0.05
P0 Weight (g)	1.5 ± 0.05	1.6 ± 0.08	1.6 ± 0.06	0.58	>0.05	1.7 ± 0.04	1.7 ± 0.06	0.16	>0.05
P7 Weight (g)	3.8 ± 0.08	3.8 ± 0.07	3.9 ± 0.11	0.14	>0.05	3.6 ± 0.10	3.5 ± 0.09	0.72	>0.05
P14 Weight (g)	6.1 ± 0.07	6.2 ± 0.05	6.3 ± 0.07	1.4	>0.05	6.3 ± 0.09	6.3 ± 0.07	0.11	>0.05
P21 Weight (g)	9.5 ± 0.04	9.7 ± 0.06	9.6 ± 0.07	1.7	>0.05	9.5 ± 0.06	9.5 ± 0.05	0.17	>0.05
Ear Detachment (day)	4.4 ± 0.30	4.1 ± 0.30	4.3 ± 0.30	0.18	>0.05	4.2 ± 0.20	4.4 ± 0.30	0.72	>0.05
Fur Appearance (day)	4.5 ± 0.30	4.3 ± 0.40	4.4 ± 0.30	0.15	>0.05	4.1 ± 0.30	4.1 ± 0.30	0.31	>0.05
Eye Opening (day)	13.1 ± 0.40	13.3 ± 0.40	12.9 ± 0.40	0.35	>0.05	12.8 ± 0.40	13.1 ± 0.30	0.87	>0.05

Table S5: Developmental milestones for F1 offspring from the paternal 0.03% and 0.015% aspartame and for F2 offspring from the paternal 0.03% lineage were compared to those for F1 and F2 offspring from the plain drinking water lineages. There were no significant differences in any of the milestones between F1 or F2 aspartame and plain drinking water lineages.
